# Psychological safety and primary care physicians’ well-being, work conditions and guideline adherence behavior: a cross-sectional study in China

**DOI:** 10.1080/16549716.2025.2484096

**Published:** 2025-04-09

**Authors:** Wenhua Wang, Jinnan Zhang, Rebecca Mitchell, Stephen Nicholas, Elizabeth Maitland, Jiao Lu, Xiaodong Liu

**Affiliations:** a School of Public Policy and Administration, Xi’an Jiaotong University, Xi’an, PR China; b Health and Wellbeing Research Unit (HoWRU), Macquarie Business School, Macquarie University, Sydney, Australia; c Newcastle Business School, The University of Newcastle, Newcastle, Australia; d Australian National Institute of Management and Commerce, Sydney, Australia; e School of Management, University of Liverpool, Liverpool, UK; f School of Public Health, Wenzhou Medical University, Wenzhou, PR China

**Keywords:** Primary health care, psychological safety, primary care physicians, guideline adherence behavior, well-being, work conditions

## Abstract

**Background:**

Health system reform initiatives have generated high-demand and created uncertain, complex practice environments, impacting the well-being, work conditions, and behavior of primary care professionals. Psychological safety is increasingly important to organizational success, particularly in highly complex work environments. Yet, few studies have examined its application among primary care professionals.

**Objectives:**

To examine the impact of psychological safety on the well-being, work conditions and guideline adherence behavior of primary care physicians (PCPs) in Community Health Centres (CHCs) in urban China.

**Methods:**

A total of 224 PCPs from 38 CHCs in four Chinese cities were recruited using convenience sampling. Research assistants conducted questionnaire surveys. Well-being was assessed through measures of depression, work-family conflict and work exhaustion, work conditions were defined by measures of professional commitment and self-directed learning, and guideline adherence behavior was measured through compliance with clinical guidelines and the provision of preventive care. Two-level hierarchical linear models were developed to analyze the impact of psychological safety.

**Results:**

Psychological safety was negatively associated with depression, work-family conflict, and work exhaustion. Psychological safety was positively associated with professional commitment and self-directed learning and also greater adherence to clinical guidelines and the increased provision of preventive care.

**Conclusions:**

This study in China confirms that a psychologically safe work environment in primary care is positively associated with improved PCPs’ well-being, enhanced professional commitment, and better adherence to clinical guidelines and preventive care provision. Given that psychological safety may be embedded in national culture, further studies should develop context-based, culturally and organizationally appropriate interventions.

## Paper context

- **Main findings:** In urban China, a psychologically safe work environment in primary care is associated with improved physician well-being, enhanced professional commitment, and better adherence to clinical guidelines and preventive care provision.
- **Added knowledge:** This study broadens the application of psychological safety in urban primary care in China, revealing its potential to transform team dynamics, improve organizational climate, and address challenges in healthcare delivery within China’s rapidly evolving primary care system.
- **Global health impact for policy and action:** Integrating psychological safety into primary 45 care improvement strategies can support physician performance and well-being, contributing to sustainable and high-quality healthcare systems globally.

## Background

Psychological safety facilitates learning behaviors when team members share the belief that the team is safe for interpersonal risk-taking, promoting feedback, sharing information, asking for help, talking about errors, and experimentation [1]. To attain shared goals and achieve organizational success, employees are expected to share information and exchange ideas with other team and organizational members [2]. In highly complex aviation, education, and manufacturing work environments, psychological safety is significantly related to information sharing and communication, learning behavior, performance, innovation and creativity, organizational commitment, work engagement, and job satisfaction [3,4]. Similarly, in healthcare settings, with complex and dynamic team environments, psychological safety may be associated with physician performance [5].

Globally, high-performing health systems rely on multidisciplinary team-based primary care delivery models [6]. Various strategies have been implemented to enhance primary care, including establishing group practices and health centers, integrating advanced technologies, expanding nurses’ roles, introducing new professionals, such as case managers, and promoting interprofessional coordination [7]. These reforms call for a fundamental transformation of the relationships and processes within existing health-care delivery systems and the restructuring of roles and responsibilities among primary care professionals. Such reform initiatives create high-demand, uncertainty and complex practice environments for primary care professionals, impacting their well-being, work conditions, and behaviors. A recent meta-analysis finds that 28.1% of primary care professionals in low- and middle-income countries experienced severe emotional exhaustion [8]. In high-income countries, job stress among general practitioners ranged from 18% to 59% [7]. During the COVID-19 pandemic, female physicians faced greater work-family conflict and exhibited more severe symptoms of depression and anxiety than their male counterparts [9].

Previous studies suggest that psychological safety reduces the risk of poor mental health outcomes among healthcare professionals, including burnout, stress and diminished well-being [10,11]. Psychological safety is also associated with higher work engagement, job performance and job satisfaction [12]; improved process adaptation [10]; and a stronger sense of belonging among team members [11,13]. Recent healthcare studies further demonstrate that psychological safety affects patient safety, interprofessional collaboration, participation in quality improvement efforts, learning from failures, and reporting adverse events [4,14]. The evidence above is mainly from secondary healthcare settings. Research on the importance of psychological safety in primary care teams, however, remains limited [15]. Given the significant differences in interprofessional dynamics, care context, and clinician well-being between primary and secondary care, research on psychological safety in primary care teams is required [16].

In urban China, community health centers (CHCs) are the main sites for primary care, providing both basic medical care and public health services [17]. CHCs are required to employ multidisciplinary teams, including clinical physicians, nurses, public health doctors and allied health professionals. Working collaboratively, these teams provide integrated care through regular case discussions, sharing responsibilities for patient management, and coordinating public health initiatives to meet the needs of the local population [17,18]. This team-based structure highlights the importance of psychological safety, as effective collaboration and open communication are essential for high-quality care deliver. As frontline providers, primary care physicians (PCPs) play a central role within these teams. Their effectiveness is directly influenced by the level of collaboration and communication within the team. Due to heavy medical and public health workloads, many PCPs in China experience low job satisfaction and high occupational burnout [19,20]. Additionally, low-quality diagnostic processes, overuse of antibiotics, poor chronic disease management [21], and high turnover intentions are common in Chinese primary care facilities [22]. Based on psychological safety theory and evidence from secondary healthcare settings, this study hypothesizes that a psychologically safe work environment can promote PCPs’ well-being (H1), improve work conditions (H2), and facilitate guideline adherence behavior (H3). Testing these hypotheses, this study examines the association between psychological safety and well-being, work conditions, and guideline adherence behavior of PCPs in Chinese urban CHCs.

## Methods

### Data source

A cross-sectional survey was conducted to assess the well-being, work conditions, guideline adherence behavior and psychological safety of PCPs in CHCs in four major Chinese cities. Shanghai, Shenzhen, Tianjin, and Jinan were chosen for their strong commitment to developing high-performing primary care systems, characterized by modern infrastructure and high-quality professionals. A convenience sampling approach was used to select 38 CHCs of varying practice size and ownership, including 10 CHCs in Shanghai, 14 in Shenzhen, 8 in Tianjin, and 6 in Jinan. At each CHC, all PCPs on duty when the researchers arrived were invited to complete a physician survey, including information on well-being, work performance, and work conditions. PCPs were included if they were directly involved in patient care and participated in regular team meetings or collaborative activities within the CHCs. PCPs were excluded if they were on temporary or extended leave, held primarily administrative roles without direct patient interaction, or belonged to CHCs undergoing major structural changes during the study period. The management teams were invited to complete an organizational survey, including information on health professionals, equipment, and health information system. Research assistants provided real-time assistance to participants to ensure complete responses. All questionnaires were reviewed on-site for completeness before submission, minimizing missing data. In total, 224 valid PCP questionnaires were collected from 38 CHCs. Ethical approval was granted by the Ethics Committee of Xi’an Jiaotong University, and all participants provided informed consent.

### Measures

#### Psychological safety

Psychological safety was measured using the mean score of seven items developed by Edmondson, each rated on a seven-point scale (1= strongly disagree, 7= strongly agree) [1]. The items included ‘If a colleague makes a mistake at work, he or she is often blamed for it’; ‘Colleagues often bring up problems and tough issues’; ‘Colleagues sometimes reject others for being different’; ‘Working with colleagues, everyone feels free to say what they want to say and try to do what they want to do’; ‘It is easy to ask colleagues for help’; ‘No one would deliberately act in a way that undermines my effort’; and ‘Working with colleagues, my unique skills and talents are valued and utilized’. The Cronbach’s alpha coefficient was 0.840.

#### Well-being indicators

Well-being was assessed using three indicators: depression, work-family conflict, and work exhaustion.

Depression was measured by the Patient Reported Outcomes Measurement Information System (PROMIS) depression four-item short form (depression SF-4) [23]. The scale assessed how participants felt during the past week regarding worthless, helpless, depressed, and hopeless, using a five-point scale (1= not at all, 5= completely). Responses to the four items were summed and standardized to a depression T-score. The Cronbach’s alpha coefficient was 0.905.

Work-family conflict was measured using a scale developed by Grzywacz and Marks to explore the negative spill-over effects between work and family [24]. Items included ‘Your job makes you feel too tired to do the things that need attention at home’; ‘Stress at work makes you irritable at home’; ‘Job worries or problems distract you when you are at home’; ‘Your job reduces the effort you can give to activities at home’. Respondents rated their agreement with each statement on a seven-point scale (1 = strongly disagree, 7 = strongly agree). The mean score of the four items represented work-family conflict. The Cronbach’s alpha coefficient was 0.907.

Work exhaustion was measured using items from the Professional Fulfilment Index (PFI) tool [25], including ‘I feel dreadful when I think about the work I have to do’; ‘I feel physically exhausted at work’; ‘I lack enthusiasm at work’; and ‘I feel emotionally exhausted at work.’ Each item was rated on a five-point scale (1= strongly disagree, 5= strongly agree). The mean score of the four items represented work exhaustion. The Cronbach’s alpha coefficient was 0.865.

#### Work conditions indicators

Work conditions were assessed using two indicators: professional commitment and self-directed learning.

Professional commitment was measured using two items on a five-point scale (1= strongly disagree, 5= strongly agree): ‘My job is ideal, and I will not give up it’, and ‘If I make a choice again, I will still choose my current job’. The mean score of the two items represented professional commitment [26,27]. The Cronbach’s alpha coefficient was 0.852.

Self-directed learning was assessed using the mean score of three items from the Qiu et al. scale in the Chinese context: ‘You actively participate in all kinds of training and strive to improve the level of business and the quality of care’; ‘You use your spare time to enrich my medical knowledge and improve my professional level’; and ‘You consciously and humbly learn from other highly skilled medical professionals’ [28]. The scale was rated on a five-point scale (1 = strongly disagree, 5 = strongly agree). The Cronbach’s alpha coefficient was 0.851.

#### Guideline adherence behavior indicators

Guideline adherence behavior was assessed using two indicators: clinical guideline adherence and preventive care provision.

Clinical guideline adherence was measured using a single item: ‘How often have you used evidence-based treatment guidelines issued by the government, medical societies, or other groups to treat patients living with chronic diseases’ [29]. PCPs rated this item on a five-point scale (1= not at all, 5= completely).

Preventive care provision was assessed using four items measuring the frequency of delivering preventive services relevant to tobacco use, alcohol use, eating habits, and physical activities [30]. For example, one representative question asks: ‘How often do you use a health risk assessment tool to identify patients who may benefit from counselling or other interventions for the tobacco use, alcohol use, eating habits, physical activity?’. PCPs rated each item on a five-point scale (1= not at all, 5= completely). The mean score of the four items represented preventive care provision. The Cronbach’s alpha coefficient was 0.855.

Other organization-level variables included organizational ownership (government-managed CHCs or hospital-managed CHCs), accreditation status (accredited by national or provincial health authorities or not), organizational size (the number of employees), percentage of health professionals, and the number of high value medical equipment (medical equipment valued over RMB10 thousand). Other physician-level variables included age, sex, education level, and employment status.

### Statistical analysis

Descriptive statistics, including the numbers (and percentages) for categorical variables and the means (and standard deviations) for continuous variables, were used to summarize the sample characteristics. Considering the non-normal distribution of the variables, Spearman’s rank correlation coefficients were used to examine the relationship between the primary variables.

Given that PCPs were nested within the CHCs, seven two-level hierarchical linear models (HLMs) were independently constructed to examine the potential association of physicians’ psychological safety with depression, work-family conflict, work exhaustion, professional commitment, self-directed learning, clinical guideline adherence, and preventive care provision. Each model controlled for PCPs’ age, sex, education level, employment status and for CHCs’ characteristics, including organizational ownership, organizational size, the number of high value medical equipment, percentage of health professionals, and accreditation status. Variables were mean-centered using centering at grand mean (CGM), excluding the dummy variables, to facilitate interpretation of regression results and to avoid problems of multicollinearity [31]. All statistical analysis was carried out using Stata 15.0.

## Results

In Table 1, more than half of PCPs were female (59.8%); the average age was 36.8 years; over 95% had completed junior college or higher education; and more than three-quarters (75.9%) had permanent employment status. Regarding the CHCs’ organizational characteristics, 60.5% were government-owned, and 39.5% were affiliated with public hospitals; over half (57.9%) were accredited by national or provincial health authorities; roughly half (52.6%) were small organizations with less than 56 employees; about half (47.4%) had over 10 items of high value medical equipment; and among CHC staff, the average percentage of health professionals was 86.6%.

**Table 1.** Description of physician and organizational characteristics.

Characteristics	*Mean* ± *SD* (range) or *n* (%)
**Physician level (*n* = 224)**	
Age	36.8 ± 7.6 (25–60)
Sex	
Male	90 (40.2)
Female	134 (59.8)
Education level	
High school or blow	9 (4)
Junior college/college	179 (79.9)
Master or above	36 (16.1)
Status	
No	54 (24.1)
Yes	170 (75.9)
**Organizational level (*n* = 38)**	
Organizational ownership	
Government	23 (60.5)
Public hospital	15 (39.5)
Accredited	
No	16 (42.1)
Yes	22 (57.9)
Organizational size	
≤35	13 (34.2)
36–55	7 (18.4)
56–100	7 (18.4)
>100	11 (29)
No. of medical equipment	
≤4	8 (21.1)
5–10	12 (31.6)
11–30	5 (13.2)
>30	13 (34.2)
Percentage of health professionals	0.9 ± 0.1 (0.6–1)

The physician data nested within 38 CHCs.

Table 2 presents the means, standard deviations, and correlations of the key PCPs’ variables. Psychological safety was negatively correlated with depression (*r* = −0.380, *p* < 0.001), work-family conflict (*r* = −0.354, *p* < 0.001), and work exhaustion (*r* = −0.519, *p* < 0.001), while positively correlated with professional commitment (*r* = 0.352, *p* < 0.001), self-directed learning (*r* = 0.443, *p* < 0.001), clinical guideline adherence (*r* = 0.257, *p* < 0.05), and preventive care provision (*r* = 0.391, *p* < 0.05).

**Table 2.** Correlation matrix for the main variables of physicians in community health centers.

	Mean	*SD*	PS	Depression	WFC	WE	PC	CGA	PCP
PS	5.254	1.097							
Depression	54.060	8.920	−0.380***						
WFC	4.171	1.644	−0.354***	0.403***					
WE	2.537	1.020	−0.519***	0.457***	0.667***				
PC	3.723	1.082	0.352***	−0.353***	−0.301***	−0.487***			
CGA	4.080	0.953	0.257**	−0.204	−0.062**	−0.206***	0.374***		
PCP	3.587	0.873	0.391**	−0.185*	−0.128**	−0.217***	0.274***	0.474***	
SDL	4.204	0.751	0.443***	−0.236**	−0.196***	−0.384***	0.548***	0.396***	0.347***

SD = standard deviation; PS = Psychological safety; WFC = Work-family conflict; WE = Work exhaustion; PC = Professional commitment; CGA = Clinical guideline adherence; PCP = Preventive care provision; SDL = Self-directed learning.

*p < 0.10; **p < 0.05; ***p < 0.001.

Table 3 presents the results of the HLM analysis on the associations between psychological safety and the seven primary variables. The intraclass correlation coefficients (ICC) of the seven null models were all greater than zero, indicating that HLM was more appropriate than the simple linear model. Psychological safety was negatively associated with depression (*Coef*. = −2.639, *p* < 0.001), work-family conflict (*Coef*. = −0.524, *p* < 0.001), and work exhaustion (*Coef*. = −0.467, *p*  < 0.001), supporting the H1 that a psychologically safe work environment promoted PCPs’ well-being. Psychological safety was positively associated with professional commitment (*Coef*. = 0.388, *p* < 0.001), and self-directed learning (*Coef*. = 0.280, *p* < 0.001), supporting the H2 that a psychologically safe work environment improved PCPs’ work conditions. Psychological safety was positively associated with clinical guideline adherence (*Coef*. = 0.228, *p* < 0.001), and preventive care provision (*Coef*. = 0.298, *p* < 0.001), supporting the H3 that a psychologically safe work environment facilitated PCPs’ guideline adherence behavior.

**Table 3.** Multilevel linear models examining the influence of psychological safety in community health centers.

Parameter	Depression		Work-family conflict		Work exhaustion		Professional commitment		Self-directed learning		Clinical guideline adherence		Preventive care provision	
	*β*	*SE*	*β*	*SE*	*β*	*SE*	*β*	*SE*	*β*	*SE*	*β*	*SE*	*β*	*SE*
**Intercept**	53.306***	3.958	4.726***	0.740	2.638***	0.411	3.900***	0.461	4.044***	0.301	4.713***	0.402	4.406***	0.374
**Key physician variable**														
Psychological safety	−2.639***	0.530	−0.524***	0.097	−0.467***	0.055	0.388***	0.061	0.280***	0.041	0.228***	0.054	0.298***	0.048
**Control variables**														
**Physician level**														
Age	−0.079	0.074	0.020	0.014	−0.007	0.008	0.033***	0.008	0.027***	0.006	0.025**	0.008	0.016**	0.007
Sex (ref = male)														
Female	2.335**	1.148	−0.085	0.208	0.008	0.121	0.012	0.130	−0.024	0.089	−0.137	0.119	0.038	0.103
Education level (ref = high school or blow)														
Junior college/college	−1.822	2.910	−0.539	0.527	−0.183	0.306	−0.522	0.330	0.127	0.227	−0.554*	0.301	−0.630**	0.260
Master or above	−2.859	3.312	−0.755	0.604	−0.376	0.347	−0.311	0.377	0.257	0.257	−0.508	0.342	−0.608**	0.299
Status (ref = no)														
Yes	−0.127	1.607	0.009	0.291	−0.044	0.169	−0.051	0.182	−0.042	0.126	−0.098	0.167	0.033	0.143
**Organizational level**														
Organizational ownership (ref = government)														
Public hospital	0.035	2.052	0.035	0.404	0.061	0.209	0.193	0.250	−0.060	0.148	0.099	0.201	0.026	0.211
Standardized CHC (ref = no)														
Yes	0.706	1.432	−0.116	0.285	−0.078	0.145	−0.045	0.176	−0.040	0.102	−0.107	0.139	−0.196	0.150
Organizational size (ref = ≤35)														
36–55	1.084	1.966	−0.043	0.391	0.059	0.199	0.174	0.242	0.073	0.141	−0.223	0.191	−0.406**	0.205
56–100	1.208	2.279	−0.146	0.457	−0.092	0.230	0.450	0.282	0.167	0.161	−0.240	0.220	−0.511**	0.241
>100	1.316	2.456	−0.204	0.503	−0.065	0.245	0.433	0.310	0.259	0.169	−0.543**	0.233	−0.293	0.268
No. of medical equipment (ref = ≤4)														
5–10	−0.515	1.954	0.143	0.384	0.145	0.199	0.091	0.238	−0.140	0.142	0.282	0.192	0.185	0.201
11–30	0.531	1.899	0.244	0.380	0.366*	0.192	0.065	0.235	0.176	0.135	0.312*	0.184	0.180	0.200
>30	0.024	2.498	0.370	0.507	0.255	0.250	−0.099	0.313	−0.131	0.174	0.807***	0.238	0.261	0.269
Percentage of health professionals	4.777	6.142	−0.044	1.216	0.179	0.623	1.304*	0.752	0.480	0.441	0.169	0.599	−0.541	0.637
***ICC***	0.060		0.097		0.084		0.101		0.029		0.086		0.145	
***AIC***	1614.852		854.199		603.986		643.371		468.483		596.131		540.878	
**Marginal *R^2^***	0.137		0.137		0.266		0.208		0.269		0.197		0.231	
**Conditional *R^2^***	0.238		0.270		0.338		0.321		0.320		0.257		0.380	

*β* = coefficient; *SE* = standard error; *ICC* = intraclass correlation coefficient; *AIC* = Akaike information criteria.

*p < 0.10; **p < 0.05; ***p < 0.001.

For other control variables, PCPs in CHCs with 11–30 items of high value medical equipment (*Coef*. = 0.312, *p* < 0.10) and those in CHCs with 30 or more items of high value medical equipment (*Coef*. = 0.807, *p* < 0.001) reported higher clinical guideline adherence scores compared with those in CHCs with less than five high value items of medical equipment. PCPs’ age was positively associated with professional commitment (*Coef*. = 0.033, *p* < 0.001), self-directed learning (*Coef*.  = 0.027, *p* < 0.001), and clinical guideline adherence (*Coef*. = 0.025, *p*  < 0.05). Female PCPs reported higher depression scores than male PCPs (*Coef*. = 2.335, *p* < 0.05).

## Discussion

Based on 224 PCPs in 38 selected CHCs across four major Chinese cities, this study confirms that a psychologically safe work environment in primary care is positively associated with improved PCPs’ well-being, enhanced professional commitment, and better adherence to clinical guidelines, as well as better preventive care provision. By applying the concept of psychological safety to primary care, our findings challenge the prevailing emphasis on resource allocation as the primary lever for improving the quality of primary care. Instead, our results highlight the psychosocial work environment as a key target for primary health-care reform, contributing to sustainable, high-quality healthcare.

There is a global consensus that strengthening primary health care is essential for addressing the demographic and health challenges of aging populations while containing costs. Healthcare reform strategies have been implemented to reorganize service delivery models, redefine team roles and responsibilities, integrate new technologies, and restructure payment models [7]. These changes have profoundly reshaped primary care, exerting substantial impacts on PCPs, including increased workloads, excessive administrative burdens, limited work-related decision autonomy, and restructured organizational support and leadership cultures [7,32]. Such challenges directly impact PCPs’ well-being, contributing to mental health issues, work-family conflict, and burnout [7–9]. Given these concerns, it is imperative to identify protective factors that contribute to the well-being of healthcare workers. Our findings suggest that a climate of psychological safety is associated with positive individual outcomes, including improved well-being. United States studies also indicate that interventions to improve the primary care practice environments may help improve PCPs’ well-being [33]. As the first study to examine this issue in Chinese CHCs, the results confirm that creating a psychologically safe work environment is a promising strategy for safeguarding PCPs’ well-being.

In Chinese CHCs, a psychologically safe work environment is positively associated with PCPs’ professional commitment and self-directed learning behavior, aligning with findings from corporate settings [3]. Social learning theory posits that learning inherently involves uncertainties and risks, which may induce stress or anxiety among team members. Psychological safety creates a supportive learning environment within the team, encouraging team members to acquire new knowledge and skills. When individuals feel secure in seeking feedback and resources to improve their capabilities, their personal learning behaviors are facilitated [34]. Further, social exchange theory suggests that employees with high perceived psychological safety experience greater autonomy, higher levels of organizational support and respect, and are more likely to reciprocate through emotional attachment and identification with their organizations and jobs [35]. This suggests that interventions fostering a psychologically safe work environment can facilitate PCPs’ learning behaviors to improve work ability and capacity to adapt effectively during complex reform processes, as well as enhancing commitment to their jobs and organizations.

We find that PCPs with high psychological safety scores are more likely to provide high quality of care with high clinical guideline adherence. This corresponds with a recent systematic literature review showing a significant relationship between psychological safety and key outcomes, such as technical team performance, continuous quality improvement, patient-centered care, as well as the successful implementation of new technologies and improvement projects [3,14]. A national survey in China reveals that 91.7% of PCPs are aware of clinical guidelines, but only 11.3% of PCPs report frequent guideline usage, underscoring a critical gap between knowledge and practice [36]. Innovative strategies are urgently needed to promote the implementation of clinical guidelines in primary care. Previous studies have primarily focused on factors at physician and guideline level, such as physician perception, commitment, self-efficacy, and guideline quality [36–42]. Our study provides a novel perspective by evidencing psychological safety, an organization level factor that is both influential and modifiable, as a key enabler of clinical guideline implementation in primary care.

To enhance psychological safety among Chinese PCPs, lessons can be drawn from international studies that emphasize leadership practices, team dynamics, and organizational strategies [5,43]. That research shows that fostering a supportive leadership style, such as transformational or inclusive leadership, is critical in cultivating an environment where healthcare professionals feel valued and respected. Leaders who actively seek input, acknowledge contributions, and model vulnerability by admitting mistakes reinforce a culture of trust and openness, which is foundational to psychological safety [15,44–46]. Team-based interventions have also proven effective. Structured activities such as regular team huddles, debriefing sessions, and cross-disciplinary learning workshops, build mutual understanding, encourage open communication, and normalize the discussion of challenges and errors [43,47–49]. We recommend primary care team professionals from diverse specialties in a shared location organize regular face-to-face interactions to enhance communication and trust [17]. Familiarity through consistent interaction further improves team dynamics and psychological safety [5]. Since frequent staff turnover and diverse communication styles pose challenges to communications and trust, we recommend promoting stability in team membership and structured communication practices to mitigate these issues and enhance mutual understanding [5]. At an organizational level, fostering a culture that encourages openness and respect reinforces psychological safety. We recommend implementing policies that encourage raising concerns as a professional duty and ensure constructive administrative responses to promote assertive communication and speaking up. When healthcare professionals feel organization values their contributions and well-being, psychological safety is significantly strengthened [5]. These strategies, though diverse, share a common goal of creating a work environment where PCPs feel empowered to voice concerns, share knowledge, and engage collaboratively. The diverse challenges facing urban Chinese primary care that vary by patient loads and dual medical and public health service responsibilities, require a tailored response specific to the needs of each CHC. Future studies should explore how these international strategies can be adapted and scaled to enhance psychological safety in China’s urban CHCs.

The strengths of this study include its multilevel design and robust measurement selection. We recruited 224 PCPs nested within 38 CHCs from four major cities with developed primary care systems and collected indicators on organization-level and physician-level characteristics. Our indicators measuring the organization-level and physician-level features have been widely used in international primary care settings and validated in the Chinese context, which ensured the validity and reliability of the study results. The convenience sampling method may restrict the generalizability of our findings. This sampling approach was necessitated by the logistical challenges posed by the COVID-19 pandemic, which limited our ability to access a broader sample. Future research should employ a probability-based sampling method and conduct longitudinal surveys across a larger number of cities to enhance the representativeness and generalizability of our findings.

## Conclusion

This study in China confirms that a psychologically safe work environment in primary care is positively associated with improved PCPs’ well-being, enhanced professional commitment, and better adherence to clinical guidelines and preventive care provision. Given that psychological safety may be embedded in national culture, further studies should identify context-based, culturally and organizationally appropriate interventions and implementations.

## Data Availability

Data underlying this article cannot be shared publicly to protect the privacy of individuals who participated in the study. Aggregate data will be shared on reasonable request to the corresponding author.
